# Cardiovascular disease risk in an urban African population: a cross-sectional analysis on the role of HIV and antiretroviral treatment

**DOI:** 10.1186/s12977-019-0497-7

**Published:** 2019-12-03

**Authors:** Alinda G. Vos, Klariska Hoeve, Roos E. Barth, Joyce Peper, Michelle Moorhouse, Nigel J. Crowther, Willem D. F. Venter, Diederick E. Grobbee, Michiel L. Bots, Kerstin Klipstein-Grobusch

**Affiliations:** 1 Julius Global Health, Julius Center for Health Sciences and Primary Care, University Medical Center Utrecht, Utrecht University, Universiteitsweg 100, 3584 CG Utrecht, The Netherlands; 2 Department of Internal Medicine & Infectious Diseases, University Medical Center Utrecht, Utrecht, The Netherlands; 3 Ezintsha, Wits Reproductive Health and HIV Institute, Faculty of Health Sciences, University of Witwatersrand, Johannesburg, South Africa; 4 Department of Cardiology, St. Antonius Hospital, Nieuwegein, The Netherlands; 5 Department of Chemical Pathology, National Health Laboratory Service and University of the Witwatersrand, Johannesburg, South Africa; 6 Division of Epidemiology and Biostatistics, School of Public Health, Faculty of Health Sciences, University of the Witwatersrand, Johannesburg, South Africa

**Keywords:** Human immunodeficiency virus, Antiretroviral therapy, Cardiovascular disease, Carotid intima-media thickness, Carotid distensibility, Sub-Saharan Africa

## Abstract

**Background:**

Life expectancy is increasing in the HIV-positive population and age-related non-communicable diseases, such as cardiovascular disease, (CVD) are seen more frequently. This study investigated to what extent HIV and antiretroviral therapy (ART) is associated with CVD risk in an urban African population.

**Methods:**

A cross-sectional study was performed in Johannesburg, South Africa, between July 2016 and November 2017. Both HIV-positive adults (ART-naïve, or on first- or second-line ART), as well as age and sex matched HIV-negative controls who were family or friends of the HIV-positive participants were included. Data were collected on demographics, cardiovascular risk factors, HIV-related characteristics, carotid intima-media thickness (CIMT) and carotid distensibility. The association between HIV, ART and CIMT and distensibility was analysed with linear regression models, adjusting for age, gender and CVD risk factors.

**Results:**

The study included 548 participants, 337 (62%) females, age 38.3 ± 9.5 years of whom 104 (19.0%) were HIV-positive, ART-naïve; 94 (17.2%) were on first-line ART; 197 (35.9%) were on second-line ART; and 153 (27.9%) were HIV-negative. Participants on second-line ART had higher CIMT and lower distensibility compared to the other groups (p < 0.001). After adjustment for age, these outcomes were similar between groups. Further adjustment for CVD and HIV-related factors did not alter the findings.

**Conclusion:**

Neither HIV nor ART was associated with CIMT or carotid distensibility in this urban African population. Longitudinal studies are needed to fully understand the relationship between HIV and CVD across different populations.

## Background

Life expectancy has increased for the human immunodeficiency virus (HIV) infected population due to the successful roll out of antiretroviral therapy (ART) [1, 2]. As a result, HIV-positive populations will increasingly experience ageing-related non-communicable diseases (NCDs) such as cardiovascular disease (CVD).

The incidence of NCDs is high in sub-Saharan Africa (SSA). In South Africa, NCDs accounted for 54.7% of all deaths in 2016 [3]. Cerebrovascular disease and ischaemic heart disease were ranked fourth and fifth for years of life lost in 2015 and diabetes was the second biggest killer after TB in 2016 [3, 4].

In high-income countries (HIC) HIV infection is associated with an increased risk of CVD of up to 50% [5, 6]. The pathophysiology is likely multifactorial and depends on chronic inflammation [7, 8], metabolic side-effects of ART and the burden of classical risk factors for CVD that is likely higher in people with HIV compared to the general population [9].

The situation for SSA is less clear, even though 70% of the world’s HIV-infected population resides in this region [10]. It is questionable whether data from HIC can be applied to SSA as the demographics of both HIV-positive populations and the general population differ substantially between these regions. Where the HIV epidemic in HIC is predominantly among white homosexual men and intravenous drug users, the majority of the HIV-infected population in SSA are black, heterosexual women [10]. Lifestyle differences are also apparent from an evaluation of the burden of classic CVD risk factors. In HIC, the percentage of people with hypertension, diabetes and smoking is higher for the HIV-positive group compared to the HIV-negative population [5, 11, 12]. In SSA these risk factors seem to be less prevalent in the HIV-positive population compared to the HIV-negative population [13–15].

The contribution made by ART to the development of CVD is a matter of debate. Although ART restores immune function, inflammatory markers do not return to normal levels, indicating ongoing low-grade inflammation, which is associated with CVD risk [16–18]. Older ART regimens evidently carry a high risk of metabolic side-effects, including lipodystrophy, altered glucose metabolism and dyslipidaemia, and have been associated with increased carotid intima-media thickness (CIMT), a marker of subclinical atherosclerosis [19]. Newer ART regimens have a lower risk of metabolic side-effects, yet markers of endothelial damage still seem to be higher in the HIV-positive population on ART compared to the HIV-negative population [20, 21]. This underlines the importance of taking ART regimens into account when evaluating CVD risk in HIV-positive individuals.

It is difficult to obtain data on the long-term effects of HIV and ART on CVD risk as many participants and years of follow-up are needed in studies using clinical endpoints such as myocardial infarction and stroke. However, alternative markers associated with future clinical CVD events are available and can be used to estimate CVD risk. CIMT is an established proxy for CVD risk in both white and black populations [22]. Arterial distensibility is a marker of subclinical atherosclerosis and is an independent predictor of cardiovascular mortality [23]. Arterial stiffening, and hence reduced distensibility, occurs as part of normal ageing and is accelerated by a number of conditions including hypertension, diabetes mellitus and renal failure [24].

Therefore, this study’s aim is to gain insight into the influence of HIV and ART on CIMT and carotid distensibility, and hence CVD risk, in an urban African population.

## Methods

### Recruitment

We performed a cross-sectional study including four groups of participants aged ≥ 18 years between July 2016 and November 2017 at Charlotte Maxeke Johannesburg Academic Hospital, Johannesburg, South Africa. All HIV-positive participants were diagnosed during routine HIV testing programmes in inner-city Johannesburg. The first group included newly diagnosed HIV-positive participants. Participants were recruited from an ongoing randomised controlled trial (RCT) comparing two new first-line ART regimens with the current standard of care first-line ART regimen [25]. Participants had HIV-1 infection and plasma HIV-1 RNA ≥ 500 copies/mL at screening and no exposure to ART. Participants were eligible for participation in our study any moment from enrolment in the RCT to a maximum follow-up duration of 36 weeks. The second group was on a first-line tenofovir containing regimen for at least 2.5 years. Participants were recruited from an RCT completed in 2016, comparing two different first line ART-regimens and included 600 HIV-positive, ART-naïve individuals in Johannesburg, South Africa, between 2012 and 2014 [26]. The third group included participants on second-line ART. They were recruited from an open label randomised study to demonstrate non-inferiority of low-dose ritonavir-boosted darunavir compared with ritonavir-boosted lopinavir-based second-line ART regimen [27]. Inclusion criteria for this trial were second-line ART for at least 48-weeks and plasma HIV-1 RNA levels < 50 copies/mL. Participants of this trial were approached for enrolment in our project during the baseline or one of the follow-up visits. All RCTs aimed to test ART in the general HIV-positive population. Exclusion criteria for these trials related to relatively rare conditions in a young population like impaired kidney and liver function and hepatitis B infection.

The fourth group consisted of HIV-negative participants recruited from the local community. The HIV-positive participants were invited to refer a family member or friend of the same age (± 5 years) and gender who was not known to be HIV-positive. All participants without a documented HIV-positive status underwent HIV testing upon enrolment according to South African Department of Health HIV testing guideline [28]. If a participant tested HIV-positive and there was no history of ART use they were assigned to the first group (HIV-positive, ART-naïve), and referred to a local clinic to initiate ART. If a participant that tested positive for HIV was found to be receiving ART, he/she was included in the group on first- or second-line ART, depending on their current ART regimen.

### Data collection

Data were collected on demographics, medical history and medication use, including HIV status and ART use, cardiovascular risk factors using a modified version of WHO-STEPs questionnaire [29] and physical activity in Metabolic Equivalent for Task (MET) minutes per week using the International Physical Activity Questionnaire [30]. Students, retirees, disabled and volunteers were considered unemployed. Being married or cohabitating was considered a stable relationship. A positive family history of CVD was defined as a history of stroke and/or heart attack of a first-degree family member (parent or sibling) before the age of 60 years. A physical examination included measurement of height, weight, waist and hip circumference. Blood pressure was measured in a seated position after a 5-min rest on the left arm and the right arm, and repeated on the arm with the highest value. The average of the second and third measurement was used for analysis. Blood was drawn for the analysis of creatinine, random glucose and lipids, and from HIV-positive participants also for HIV viral load. Urine was collected for analysis of albumin to creatinine ratio. An accredited laboratory analysed blood and urine samples. For the HIV-positive, ART-naïve participants who were recruited from the ongoing RCT, and for participants on second-line ART, laboratory data of the clinical trial visit closest to the visit at our study site was used. For participants on first-line ART, HIV viral load and CD4+ cell count were retrieved from the last RCT study visit.

### Outcome measurement

CIMT was measured in all participants after a 15 min rest. We used a Siemens Acuson p500 ultrasound (Siemens Healthcare (Pty) Ltd, South Africa) and scans were obtained in B-mode with a ≥ 7.0 MHz linear probe. The near wall and far wall of the common carotid artery (CCA) was measured at three standardised angles at both the right and left side using the Meijer’s Arc [31]. The far wall of the carotid bulb on the right and left side was captured at the best visible angle. A 5-s video-clip of both arteries was made with the transducer positioned in line with the CCA one centimetre from the carotid bifurcation. CIMT was measured semi-automatically with the Artery Measurement System software (Chalmers University, Götenburg, Sweden) and adjusted manually if needed [32]. Analyses were done in batch with a uniform reading protocol. CIMT reading included mean and maximum thickness of the intima-media layer of the near and far wall across all six angles of the CCA (mean CCA intima-media thickness (IMT) and max CCA-IMT), and the maximum IMT at the carotid bulb left and right (max bulb-IMT). A mean CCA-IMT of > 1.0 mm at any of the measured angles was considered as a plaque [33].

For carotid distensibility, approximately 100 discrete measurement points were used to obtain the maximum (LDmax) and minimum (LDmin) carotid lumen diameter in a 10 mm segment within 20 mm from the bifurcation. One reader read the CIMT images and two readers analysed carotid distensibility clips. The readers were blinded to the HIV status and treatment group. Inter-reader agreement for the clips was 0.90 (95% CI 0.62–0.96) using the intra-class correlation coefficient with a two-way random-effects model.

Pulse pressure (PP) was calculated by subtracting the diastolic blood pressure from the systolic blood pressure. The carotid distensibility was calculated according to the following formula:

$$Distensibility = \frac{{\left( {\frac{{2\left( {LDmin - LDmax} \right)}}{LDmin}} \right)}}{PP}$$

Lower values reflect a stiffer carotid artery. Distensibility was standardized according to units in 10^−6^ * New-tons^−1^ * metres^2^ [34]. Measurements of the right CCA were used for analysis [35–37].

The study was approved by the Human Ethics Research Committee of the University of the Witwatersrand (ethics clearance number M160130) and performed conform the Declaration of Helsinki. All participants provided written, informed consent prior to participation.

### Statistical analysis

Baseline characteristics were presented for each of the four groups as mean [standard deviation (SD)], median [interquartile range (IQR)] or frequency count [percentage (%)] as appropriate. First, CIMT outcomes and carotid distensibility were compared across the groups using the Kruskal–Wallis test with *post*-*hoc* testing using a Bonferroni correction. Second, the relationship of HIV and ART status with mean and max CCA-IMT, max bulb-IMT and carotid distensibility was analysed using linear regression models. The first model included all groups using the HIV-negative group as reference group with no adjustments; the second model was adjusted for age; the third model was adjusted for age and sex; and the fourth model was further adjusted for CVD risk factors that were shown in the literature to be related to CIMT, namely systolic blood pressure, body mass index, LDL cholesterol, HDL cholesterol, glucose and current smoking [31]. An additional analysis was performed using the same method including the HIV-positive participants only, using the ART-naïve group as the reference to assess the contribution of HIV related factors, namely CD4+ cell count, HIV viral load and duration since HIV diagnosis. Third, models were run separately for men and women, and a possible interaction between HIV status and age was investigated by adding an interaction term to the models. A two-sided p < 0.05 was considered as statistically significant. Statistical analyses were performed using IBM SPSS Statistics Version 25 (SPSS, Chicago, Illinois, USA).

## Results

In total, 548 participants were included: 153 HIV-negative controls; 104 newly diagnosed HIV-positive ART-naïve participants; 94 participants with HIV on stable first-line therapy; and 197 participants on stable second-line ART (Table 1). All except one were Black African, the majority were women (n = 337, 61.5%) and the mean age was 38.3 (SD 9.5) years. Overall, 38.4% completed matric or university, and most participants were single. Employment varied significantly with the highest employment rate for participants on first-line ART (82.8%) and the lowest employment rate for the HIV-negative controls (32.9%). Participants on second-line ART were older, more likely to be women, and weighed more than the other participants. Participants on first-line ART knew their HIV diagnosis for about 4 years, and participants on second-line ART for approximately 9 years.

**Table 1:** Characteristics of the study population

	HIV-negative		HIV-positive	
		ART-naïve	First-line ART	Second-line ART
	n = 153	n = 104	n = 94	n = 197
Demographics and socio-economic status				
Age (years)	34.6 (10.6)	33.9 (8.4)	36.9 (6.4)	43.0 (8.0)
Women, *n*(%)	76 (49.7)	65 (62.5)	57 (60.6)	139 (70.6)
Highest level of education (n = 542), *n*(%)				
Primary school or less	12 (7.9)	13 (12.7)	10 (10.8)	27 (13.8)
Secondary school completed	74 (48.7)	51 (50.0)	51 (54.8)	96 (49.2)
More than secondary school	66 (43.4)	38 (37.2)	32 (34.4)	72 (37.0)
Work status (n = 545), *n*(%)				
Employed	50 (32.9)	55 (53.4)	77 (82.8)	132 (67.0)
Unemployed	102 (76.1)	48 (46.6)	16 (17.2)	65 (33.0)
Marital status (n = 545), *n*(%)				
Married or in a stable relationship	31 (20.4)	15 (14.6)	36 (38.7)	75 (38.1)
Single	121 (79.6)	88 (85.4)	57 (61.3)	122 (61.9)
Cardiovascular risk profile				
Family history of CVD (n = 547), *n*(%)	7 (4.6)	2 (1.9)	4 (4.3)	21 (10.7)
Physical activity, MET min/week	1200 (720–1920)	1198 (868–1693)	834 (445–2092)	984 (480–1688)
Current smokers (n = 113), *n*(%)	56 (37.1)	27 (26.2)	13 (13.8)	17 (8.6)
Packyears for current smokers, (median, IQR)	3.6 (1.3–7.3)	3.9 (2.2–6.8)	2.2 (0.7–3.6)	7.4 (2.3–12.1)
Former smokers (n = 34), *n*(%)	6 (4.0)	7 (6.8)	7 (7.4)	14 (7.1)
Never smokers (n = 398), *n*(%)	89 (58.6)	69 (67.0)	74 (78.7)	166 (84.3)
Systolic blood pressure (mmHg) (n = 547)	123.8 (18.5)	119.5 (15.1)	124.2 (17.2)	121.5 (18.8)
Diastolic blood pressure (mmHg) (n = 547)	77.8 (12.8)	75.2 (8.8)	81.4 (10.4)	77.8 (11.5)
Body mass index (kg/m^2^) (median, IQR)	24.5 (21.2–28.9)	23.6 (20.9–27.0)	24.2 (21.3–29.1)	26.3 (22.9–32.0)
< 18.5 kg/m^2^, *n*(%)	9 (5.9)	7 (6.7)	3 (3.2)	4 (2.0)
18.5–24.9 kg/m^2^, *n*(%)	74 (48.4)	57 (54.8)	49 (52.1)	76 (38.6)
25.0–29.9 kg/m^2^, *n*(%)	36 (23.5)	25 (24.0)	20 (21.3)	50 (25.4)
≥ 30.0 kg/m^2^, *n*(%)	34 (22.2)	15 (14.4)	22 (23.4)	67 (34.0)
Waist-to-hip ratio (n = 547)	0.85 (0.06)	0.85 (0.06)	0.85 (0.09)	0.87 (0.06)
Total-C (mmol/L) (n = 522)	4.1 (0.9)	3.9 (0.8)	4.4 (1.1)	4.8 (0.8)
HDL (mmol/L) (n = 522)	1.4 (0.5)	1.2 (0.5)	1.4 (0.4)	1.4 (0.4)
LDL (mmol/L) (n = 522)	2.2 (0.7)	2.3 (0.7)	2.5 (0.9)	3.1 (0.8)
TG (mmol/L), (n = 519), median (IQR)	1.0 (0.7–1.3)	0.9 (0.7–1.2)	1.0 (0.8–1.5)	1.2 (0.9–1.7)
Total-C:HDL ratio (n = 522)	3.1 (0.9)	3.4 (1.0)	3.4 (1.1)	3.7 (1.1)
Random glucose (mmol/L) (n = 514)	4.9 (1.0)	4.5 (0.9)	5.0 (0.9)	4.8 (1.6)
Creatinine clearance (mL/min) (n = 523)	119.7 (32.3)	122.9 (33.9)	121.9 (33.2)	120.7 (38.3)
Urine protein present (n = 164), *n*(%)	N/A	7 (9.1)	2 (2.3)	N/A
Albumin-to-creatinine ratio, (n = 439), median (IQR)	0.8 (0.4–1.5)	0.9 (0.5–1.9)	0.7 (0.5–1.5)	0.9 (0.5–1.8)
HIV/ART characteristics (n = 395)				
CD4-cell count (cells/mm^3^) (n = 350), median (IQR)	–	281^a^ (192–401)	414 (279–575)	619 (430–798)
Viral load (cp/mL) (n = 380), *n*(%)				
< 50	–	12 (11.8)	81 (91.0)	180 (95.2)
50–1000	–	10 (9.8)	3 (3.4)	4 (2.1)
> 1000	–	80 (78.4)	5 (5.6)	5 (2.6)
Years since diagnosis (n = 393), median (IQR)	–	0.0 (0.0–0.1)	3.9 (3.1–6.0)	9.0 (7.0–12.4)
Time on ART (n = 355), months, median (IQR)	–	0 (0.0–0.0)	39 (35–48)	96 (72–126)

Outcomes in mean with SD unless otherwise specified

*AP* angina pectoris, *ART* antiretroviral therapy, *CVA* cerebrovascular accident, *CVD* cardiovascular disease, *HDL-C* high-density lipoprotein cholesterol, *HIV* human immunodeficiency virus, *IQR* interquartile range, *LDL-C* low density lipoprotein cholesterol, *MET* metabolic equivalent of Task, *MI* myocardial infarction, *N/A* not available, *SD* standard deviation, *Total-C* total cholesterol, *TG* triglycerides

^a^Nadir CD4-cell count

CCA-IMT was available for 534 (97.4%) participants, bulb-IMT for 474 (86.5%), and carotid distensibility for 514 (93.8%) participants. Mean and max CCA-IMT and max bulb-IMT were significantly higher and carotid distensibility was significantly lower for participants on second-line ART compared to the other groups (Table 2a and b). There were only a few participants with plaque in the CCA. When CCA-IMT and distensibility outcomes were adjusted for age, differences between the groups disappeared. Further adjustment for CVD risk factors did not change the magnitude and direction of the relation between HIV, ART and mean or max CCA-IMT or carotid distensibility (see Table 3a and b for the models for mean CCA-IMT and carotid distensibility). Following multivariable adjustment age (β = 0.006, p < 0.001), systolic blood pressure (β = 0.000, p = 0.01) and LDL cholesterol (β = 0.009, p = 0.03) were associated with mean CCA-IMT. The same factors contributed to max CCA-IMT (data not shown).

**Table 2:** (a) CIMT and (b) carotid distensibility per group

	HIV-negative		HIV-positive		*p*
		ART-naïve	First-line ART	Second-line ART	
	n = 152	n = 104	n = 87	n = 191	
(a) CIMT (mm)					
CCA mean (mm)	0.504 (0.477–0.562)	0.504 (0.470–0.560)	0.521 (0.488–0.564)	0.564 (0.512–0.655)	< 0.001^†^
CCA max (mm)	0.575 (0.525–0.669)	0.575 (0.523–0.651)	0.594 (0.554–0.653)	0.667 (0.598–0.756)	< 0.001^†^
Plaque^a^	0	1 (1.0%)	0	5 (2.6%)	0.08

	n = 133	n = 98	n = 80	n = 163	
Bulb max (mm)	0.686 (0.583–0.802)	0.673 (0.542–0.824)	0.717 (0.579–0.797)	0.809 (0.698–0.971)	< 0.001^†^

	n = 148	n = 100	n = 82	n = 184	
(b) Carotid distensibility (10^−6^ N^−1^ m^2^)					
Pulse pressure (mmHg)	45.5 (38.5–51.0)	42.5 (39.0–50.0)	41.5 (34.5–50.6)	41.5 (36.6–49.5)	0.06
LD min right CCA (mm)	5.765 (5.331–6.160)	5.813 (5.444–6.102)	5.699 (5.423–6.098)	5.706 (5.421–6.155)	0.932
LD diff right CCA (mm)	0.633 (0.489–0.784)	0.618 (0.475–0.769)	0.566 (0.451–0.709)	0.530 (0.434–0.632)	< 0.001^‡^
Distensibility (10^**−6**^ N^−1^ m^2^)	36.96 (27.03–48.00)	37.82 (26.75–50.12)	35.91 (27.45–45.04)	31.68 (25.93–40.85)	0.009^§^

Outcomes in median with IQR

The p-value is based on the Kruskal-Wallis test with post-hoc testing using a Bonferroni correction

*ART* anti-retroviral therapy, *CCA* common carotid artery, *CIMT* carotid artery intima-media thickness, *LD* luminal diameter, *mm* millimeter, *IQR* interquartile range

†p<0.001 for second-line ART vs ART-naïve, second-line ART vs first-line ART and second-line ART vs HIV-negative

^‡^p = 0.002 for second-line ART vs ART-naïve and p < 0.001 for second-line ART vs HIV-negative

^§^p = 0.025 for second-line ART vs ART-naïve and p < 0.036 for second-line ART vs HIV-negative

^a^Mean CCA thickness > 1.0 mm at any angle

**Table 3:** HIV and ART status on mean (a) CCA intima-media thickness and (b) carotid distensibility

	HIV-negative			HIV-positive			
		ART-naïve	*p*	First-line ART	*p*	Second-line ART	*p*
a) HIV and ART status on mean CCA intima-media thickness (n = 534)							
Model 1	REF	− 0.015 (− 0.038 to 0.007)	0.178	− 0.001 (− 0.025 to 0.023)	0.239	0.052 (0.033 to 0.071)	< 0.001
Model 2	REF	− 0.010 (− 0.028 to 0.007	0.239	− 0.012 (− 0.031 to 0.006)	0.187	0.000 (− 0.016 to 0.016)	0.963
Model 3	REF	− 0.009 (− 0.027 to 0.008)	0.287	− 0.012 (− 0.030 to 0.007)	0.219	0.002 (− 0.015 to 0.018)	0.854
Model 4^a^	REF	− 0.011 (− 0.029 to 0.008)	0.253	− 0.012 (− 0.032 to 0.007)	0.224	− 0.005 (− 0.023 to 0.013)	0.557
b) HIV and ART status on carotid distensibility (10^−6^ N^−1^ m^2^) (n = 514)							
Model 1	REF	1.314 (− 2.707 to 5.335)	0.521	− 1.365 (− 5.641 to 2.911)	0.531	− 4.315 (− 7.745 to − 0.885)	0.014
Model 2	REF	0.675 (− 2.939 to 4.290)	0.714	0.280 (− 3.573 to 4.134)	0.886	2.387 (− 0.916 to 5.690)	0.156
Model 3	REF	0.470 (− 3.165 to 4.104)	0.800	0.107 (− 3.759 to 3.973)	0.957	2.019 (− 1.353 to 5.391)	0.240
Model 4^b^	REF	− 1.567 (− 4.891 to 1.757)	0.355	0.268 (− 3.351 to 3.888)	0.884	0.278 (− 3.046 to 3.601)	0.870

β-coefficient with 95% CI

Model 1 unadjusted

Model 2 adjusted for age

Model 3 adjusted for age and sex

Model 4 adjusted for age, sex, systolic blood pressure, body mass index, LDL cholesterol, HDL cholesterol, glucose and current smoking

*ART* antiretroviral therapy, *CCA* common carotid artery, *CI* confidence interval, *HIV* human immunodeficiency virus, *REF* reference

^a^n = 37 (6.9%) participants were excluded from this model due to missing data in one or more covariates

^b^n = 35 (6.8%) participants were excluded from this model due to missing data in one or more covariates

Max bulb-IMT was lower in HIV-positive ART-naïve participants compared to HIV-negative participants (β = − 0.070, p = 0.03) following multivariable adjustment and participants on first-line ART tended to have a lower max bulb-IMT (β = − 0.065, p = 0.07). Max bulb-IMT did not differ between participants on second-line ART and HIV-negative controls (p = 0.20) (Additional file 1: Table S1).

Table 4 shows the mean CCA-IMT and carotid distensibility for the HIV-positive participants only. After adjustment including HIV-related factors, ART was not related to mean CCA-IMT, max CCA-IMT, max bulb-IMT nor carotid distensibility.

**Table 4:** ART status on mean (a) CCA intima-media thickness and (b) carotid distensibility

	ART-naïve	First-line ART	*p*	Second-line ART	*p*
(a) ART status and mean CCA intima-media thickness					
Model 1	REF	0.014 (− 0.011 to 0.039)	0.259	0.067 (0.046 to 0.088)	< 0.001
Model 2	REF	− 0.002 (− 0.022 to 0.018)	0.829	0.009 (− 0.010 to 0.028)	0.341
Model 3	REF	− 0.002 (− 0.023 to 0.018)	0.826	0.011 (− 0.008 to 0.029)	0.272
Model 4^a^	REF	− 0.012 (− 0.038 to 0.014)	0.360	− 0.016 (− 0.046 to 0.014)	0.291
(b) ART status and carotid distensibility (10^−6^ N^−1^ m^2^) (n = 366)					
Model 1	REF	− 2.679 (− 7.221 to 1.864)	0.247	− 5.629 (− 9.417 to − 1.841)	0.004
Model 2	REF	− 0.565 (− 4.816 to 3.686)	0.794	1.166 (− 2.755 to 5.087)	0.559
Model 3	REF	− 0.562 (− 4.819 to 3.694)	0.795	1.107 (− 2.855 to 5.070)	0.583
Model 4^b^	REF	1.090 (− 3.698 to 5.879)	0.654	− 0.042 (− 5.646 to 5.563)	0.988

β-coefficient with 95% CI

Model 1 unadjusted

Model 2 adjusted for age

Model 3 adjusted for age and sex

Model 4 adjusted for age, sex, systolic blood pressure, body mass index, LDL cholesterol, HDL cholesterol, glucose, current smoking, CD4+ cell count, HIV viral load and duration since HIV infection

*ART* antiretroviral therapy, *CCA* common carotid artery, *IMT* intima media thickness

^a^n = 57 (14.9%) participants were excluded from this model due to missing data in one or more covariates

^b^n = 56 (15.3%) participants were excluded from this model due to missing data in one or more covariates

Finally, men and women were analysed separately and the same relations were observed (data not shown). There was no indication that the effect of HIV on CIMT or carotid distensibility differed between younger and older participants as the interaction term between age and HIV was not statistically significant.

## Discussion

HIV and ART were not related to increased CIMT and reduced carotid distensibility and hence CVD risk in this well-characterised African population. Participants on second-line ART had a higher CIMT and lower carotid distensibility, but this was explained by participants being older in this group. Max bulb-IMT was lower for HIV-positive ART-naïve participants than for HIV-negative participants. Additional correction for HIV-related factors such as CD4+ cell count, HIV viral load and duration since HIV diagnosis did not change the direction or magnitude of the relationship between HIV, ART and CVD risk.

There is no consensus in the literature on the relationship between HIV and CIMT. Several studies, all conducted outside SSA, comparing HIV-positive individuals, whether or not on ART, to HIV-negative individuals found a higher CIMT for the HIV-infected group, indicating a higher CVD risk [35, 38–41]. Furthermore, HIV infection seems to be related to CIMT as the level of viraemia influences CIMT, with a lower CIMT for people with an undetectable viral load as compared to people with low-level viraemia [42]. However, other studies did not find a difference in baseline CIMT between HIV-positive and HIV-negative participants [43–45].

The association between HIV and carotid distensibility has previously been addressed in two studies conducted in HIC. The first included 77, predominantly white, males (median age 42.3 years), including both ART-naïve patients and patients on ART [35]. The second study included 2789 participants, half of them Afro-Americans; the average age was 44.3 years [36]. Both observed HIV to be associated with a significantly lower carotid distensibility, and hence higher arterial stiffness.

The influence of ART on CIMT is also debated. Some studies showed a higher CIMT for people on ART [40, 41], whereas other studies showed no difference in CIMT between HIV-positive, both untreated and ART-treated groups [45, 46]. The class of antiretrovirals may influence the association with CIMT [47, 48]. ART use was also not consistently associated with arterial stiffness [35, 36].

Results presented in the current analysis are in line with those from other, smaller, studies conducted in SSA. Gleason et al. [49], Fourie et al. [46] and Mosepele et al. [18] all compared HIV-positive to HIV-negative participants in Ethiopia, South Africa and Botswana respectively, and observed no increase in CIMT based on HIV or ART status. Fourie et al. [46], even found a lower CCA-IMT for the HIV-positive compared to the HIV-negative group. We found a lower max bulb-IMT, but not CCA-IMT, for HIV-positive participants not yet on ART compared to HIV-negative participants. The reason for this finding is not known, however it is interesting to note that previous studies in SSA have shown a lower prevalence of classic CVD risk factors in HIV-positive compared to HIV-negative subjects [13–15]. Our findings should be confirmed in future research. Schoffelen et al. [50] in a cross-sectional study performed in South-Africa in HIV-positive participants, showed a relationship of classic CVD risk factors to CIMT, but not for HIV-related factors. In accordance with this, none of the HIV-related factors in our study were associated with CIMT. These findings suggest that neither HIV nor ART increase CIMT in a Black African population.

To interpret the conflicting findings on the relationship between HIV and CIMT the following arguments should be considered. First, studies vary in sample size, and a small sample size is more likely to result in biased findings. Second, the duration of uncontrolled HIV infection should be taken into account as this was strongly related to CVD [51] and, to a lesser extent, CIMT [38, 40]. Third, the selection of the HIV-negative control group can lead to biased findings as controls are often matched on age and gender only and not on socio-economic status. The HIV-positive groups in HIC represent a group of people with a specific lifestyle and higher CVD risk than the general population. Although studies correct for CVD risk factors there may be unmeasured factors that result in a higher CVD risk, and hence CIMT, in the HIV-positive population. Furthermore, our study included mainly women of premenopausal age, who are known to have a low CVD risk in general. CVD risk and potential differences in CVD risk between HIV-positive and HIV-negative participants may become more pronounced in post-menopausal women. Finally, when interpreting our results the urban location of our study should be considered. It would be worthwhile to repeat our study in a rural community as differences in lifestyle could result in distinct CVD risk profiles, although we do not foresee how this would influence the effect of HIV and ART on CVD risk or CIMT.

We did not find an interaction between HIV and age on CIMT. This is in contrast to a recent pooled analysis of CIMT across five cohorts of HIV-positive and HIV-negative individuals. In this analysis the relationship between HIV and CIMT was found to be age dependent: for the age group 30–49 years no association was seen between HIV and CIMT, whereas from the age of 50 years HIV infection was associated with lower CIMT than in the HIV-negative group [52]. Traditional CVD risk factors were observed to be the main drivers of CIMT and this effect increased with age [52]. In our study the number of participants over the age of 50 years was relatively small (10.9%), which might explain why we did not find an age-dependent relation between HIV and CIMT.

In summary, our findings do not support the view that CVD risk is increased in the HIV-infected population compared to the non-infected population. The question remains whether CIMT and carotid distensibility are appropriate surrogate markers to detect a difference in CVD risk in a relatively young and otherwise healthy population. Two studies in SSA could not identify an association between CIMT and elevated markers of endothelial damage s-VCAM and s-ICAM [21, 46], indicating that there is endothelial damage even when CIMT is still normal.

To date, most research in SSA using clinical endpoints such as myocardial infarction and stroke observed an increased risk for the HIV-infected population [53–55]. A recent meta-analysis using national disability-adjusted life-year estimates for cardiovascular disease found that people with HIV are twice as likely to develop cardiovascular disease as compared to the non-infected population, and that this burden was higher in SSA than in HIC [56], possibly indicating that CVD risk is increased in HIV infection in SSA.

Major strengths of our study are the large sample size; the use of an HIV-negative control group; the comparison between HIV-infected participants not yet on treatment, on stable first-line and stable second-line ART; and the extensive, standardised assessment of CVD risk.

However, some limitations need to be recognised. We used a full case analysis and this resulted in 15% missing data in the models assessing the influence of HIV-related characteristics. Assuming data were missing completely at random, any bias is likely to be small. HIV-positive participants were recruited from clinical trials using stringent inclusion criteria while the HIV-negative controls were recruited from the community. This may lead to an underestimation of the CVD risk in the HIV-positive participants; although impaired kidney function was the only CVD risk factor that was used as an exclusion criterion in the RCTs. The high unemployment rate in the HIV-negative group indicates that unemployed people were keener to participate than people who would have had to take leave from work. In addition, this group had slightly more men than the HIV-positive groups and a high percentage of current smokers. Therefore, it might be possible that the HIV-negative group does not precisely reflect CVD risk in the general, non-HIV infected population precisely. Finally, we could not correct for HIV severity at start of ART as we only had nadir CD4+ cell count for the newly diagnosed HIV-positive participants.

## Conclusion

In this urban South African cohort, neither HIV nor ART were associated with CVD risk as assessed by CIMT and carotid distensibility. The question remains whether underlying immune activation and endothelial damage have not yet resulted in increased CIMT and reduced carotid distensibility in this young population. Future research using clinical endpoints such as stroke and myocardial infarction is needed to gain further insight into the role of HIV and ART on CVD risk in the SSA context. Routine HIV care should focus on prevention and treatment of CVD risk factors known to be major drivers of CVD risk.

## Supplementary information

**Additional file 1: Table S1.** Influence of HIV and ART status on max bulb intima-media thickness.

## Data Availability

The datasets used and/or analysed during the current study are available from the corresponding author on reasonable request.
