# Supplementary material for: Cardiovascular disease risk in an urban African population: a cross-sectional analysis on the role of HIV and antiretroviral treatment
Source: Retrovirology. 2019 Dec 3;16:37. doi: 10.1186/s12977-019-0497-7 (PMC6889610; doi:10.1186/s12977-019-0497-7)
Supplement: Supplementary file 1 — Additional file 1: Table S1. Influence of HIV and ART status on max bulb intima-media thickness. [file 12977_2019_497_MOESM1_ESM.docx]

| **Additional file 1.** | | **Influence of HIV and ART status on max bulb intima-media thickness.** | | | | | |
| --- | --- | --- | --- | --- | --- | --- | --- |
|  | HIV-negative |  |  | HIV-positive |  |  |  |
|  |  | ART-naive | *p* | First-line ART | *p* | Second-line ART | *p* |
| Model 1 | REF | -0.068  (-0.147 – 0.010) | 0.088 | -0.033  (-0.017 – 0.050) | 0.433 | 0.114  (0.045 – 0.183) | 0.001 |
| Model 2 | REF | -0.069  (-0.136 – -0.002) | 0.043 | -0.072  (-0.143 – 0.000) | 0.049 | -0.043  (-0.106 – 0.020) | 0.182 |
| Model 3 | REF | -0.068  (-0.135 – 0.000) | 0.049 | -0.071  (-0.142 – 0.001) | 0.054 | -0.041  (-0.105 – 0.024) | 0.216 |
| Model 4^1^ | REF | -0.071  (-0.135 – -0.007) | 0.030 | -0.066  (-0.135 – 0.004) | 0.064 | -0.044  (-0.109 – 0.022) | 0.193 |
| Model 1 unadjusted  Model 2 adjusted for age  Model 3 adjusted for age and sex  Model 4 adjusted for age, sex, systolic blood pressure, body mass index, LDL cholesterol, HDL cholesterol, glucose and current smoking  1. n = 31 (6.5%) participants were excluded from this model due to missing data in one or more covariates | | | | | | | |
